# Late-life decompensation in longstanding obsessive compulsive disorder: a psychiatric prodrome of dementia with Lewy bodies? A case report

**DOI:** 10.1093/omcr/omag115

**Published:** 2026-07-08

**Authors:** R E Agnew, F Garcia, C Barton, S Kearney, J P M Kane

**Affiliations:** Southern Health and Social Care Trust, 68 Lurgan Rd, Portadown, Craigavon BT63 5QQ, Northern Ireland (NI), United Kingdom; Belfast Health and Social Care Trust, Royal Victoria Hospital, 274 Grosvenor Road, Belfast, BT12 6BA, NI, United Kingdom; Belfast Health and Social Care Trust, Royal Victoria Hospital, 274 Grosvenor Road, Belfast, BT12 6BA, NI, United Kingdom; Belfast Health and Social Care Trust, Royal Victoria Hospital, 274 Grosvenor Road, Belfast, BT12 6BA, NI, United Kingdom; Belfast Health and Social Care Trust, Royal Victoria Hospital, 274 Grosvenor Road, Belfast, BT12 6BA, NI, United Kingdom; Centre for Public Health, Queen’s University Belfast, Institute of Clinical Sciences B, Royal Victoria Hospital, Belfast, BT12 6BA, NI, United Kingdom

**Keywords:** dementia, neurodegenerative disease, synucleinopathies, Lewy body dementia, neuropsychiatry, obsessive compulsive disorder

## Abstract

Psychiatric-onset dementia with Lewy bodies (poDLB) is a recognised but poorly characterised prodromal phenotype. The existing literature focuses almost entirely on *de novo* psychiatric presentations; decompensation of pre-existing primary psychiatric illness as a manifestation of poDLB has not previously been described. We present a case of a patient with a near-lifelong history of severe obsessive compulsive disorder (OCD), decompensation of which in her early 70s was later recognised as a harbinger of dementia with Lewy bodies (DLB). Escalating OCD symptoms, emergent parkinsonism, and cognitive fluctuations culminated in a probable DLB diagnosis within one year of psychiatric presentation, supported by significantly abnormal dopamine transporter imaging. Recognising decompensation of longstanding primary psychiatric illness as a potential manifestation of poDLB has important implications for timely diagnosis, safe prescribing (particularly neuroleptics) and early biomarker-driven referral.

## Introduction

Dementia with Lewy Bodies (DLB), the second most prevalent neurodegenerative dementia, is associated with increased mortality and caregiver burden [1, 2]. Diagnostic criteria require the presence of two or more core clinical features (visual hallucinations, parkinsonism, cognitive fluctuations and REM sleep behaviour disorder (RBD)), or one core feature with an abnormal biomarker [1].

Three prodromal DLB phenotypes have been proposed: mild cognitive impairment-onset (MCI-LB), delirium-onset DLB, and psychiatric-onset DLB (poDLB) [3]. Compared with MCI-LB, poDLB is poorly characterised; depressive and psychotic episodes are the most commonly described presentations, with obsessive compulsive disorder (OCD) reported in only two cases to date [4, 5]. Here we present the first case of decompensation of longstanding stable OCD as a manifestation of poDLB.

## Case report

This patient was first diagnosed with severe OCD in her early 20s, shortly after the conclusion of an emotionally abusive relationship. She maintained skilled employment throughout her adult life with community psychiatry and family support, and was independent in all activities of daily living (ADLs). Compulsive spending and hoarding behaviour in her early 50s necessitated family financial support, but following stabilisation she was discharged from community services.

In her early 70s she was re-referred to psychiatry of old age (POA) by her general practitioner (GP), reporting an 18-month history of deteriorating mood. The GP had initiated risperidone 1 mg OD, identified a resting tremor, and referred to neurology. POA review found escalating compulsive behaviours, mild re-emergence of sexual and violent obsessions, moderate depressive symptoms, multiple falls, and mild bradykinesia and rigidity on activation manoeuvre. Fluoxetine was increased to 80 mg OD and risperidone tapered and stopped given concern for drug-induced parkinsonism. A DaTSCAN was requested.

Over the following three months mental state deteriorated, apparently accelerated by risperidone cessation. Sexualised mental images (the patient’s recognised relapse signature) and egodystonic violent obsessions escalated, with suicidal ideation precipitating voluntary inpatient admission. DaTSCAN, performed prior to admission while off risperidone, demonstrated significant bilateral putaminal blunting. Risperidone 0.5 mg OD was restarted with improvement in obsessional symptoms, though gait disturbance and bradykinesia were exacerbated. A movement disorder neurologist diagnosed idiopathic Parkinson’s disease and cautiously commenced co-beneldopa 62.5 mg OD, which immediately exacerbated violent obsessions and hoarding, leading to cessation after two days.

The patient expressly wished that OCD treatment take precedence over motor function. A regimen of risperidone 0.5 mg OD, pregabalin 25 mg BD, and fluoxetine 80 mg OD proved most effective for OCD symptoms. Fluctuating cognitive impairment was noted during admission, with MMSE scores of 25–29, points typically lost on memory and concentration domains, and evidence of executive dysfunction. MCI was diagnosed.

The patient was discharged to a residential care home as motor symptoms precluded independent living. OCD symptoms stabilised but cognitive and functional decline progressed; deficits in concentration and anterograde memory, with evidence of executive dysfunction, have all shown significant progression since initial presentation. Instrumental ADLs, including managing appointments, medication and finances, are no longer consistently intact. In the presence of spontaneous parkinsonism, fluctuating cognition, and an abnormal DaTSCAN within one year of cognitive impairment onset, a probable DLB diagnosis was made. Eighteen months post-admission, co-beneldopa was successfully re-trialled to 125 mg three times daily.

## Discussion

**Figure 1:**
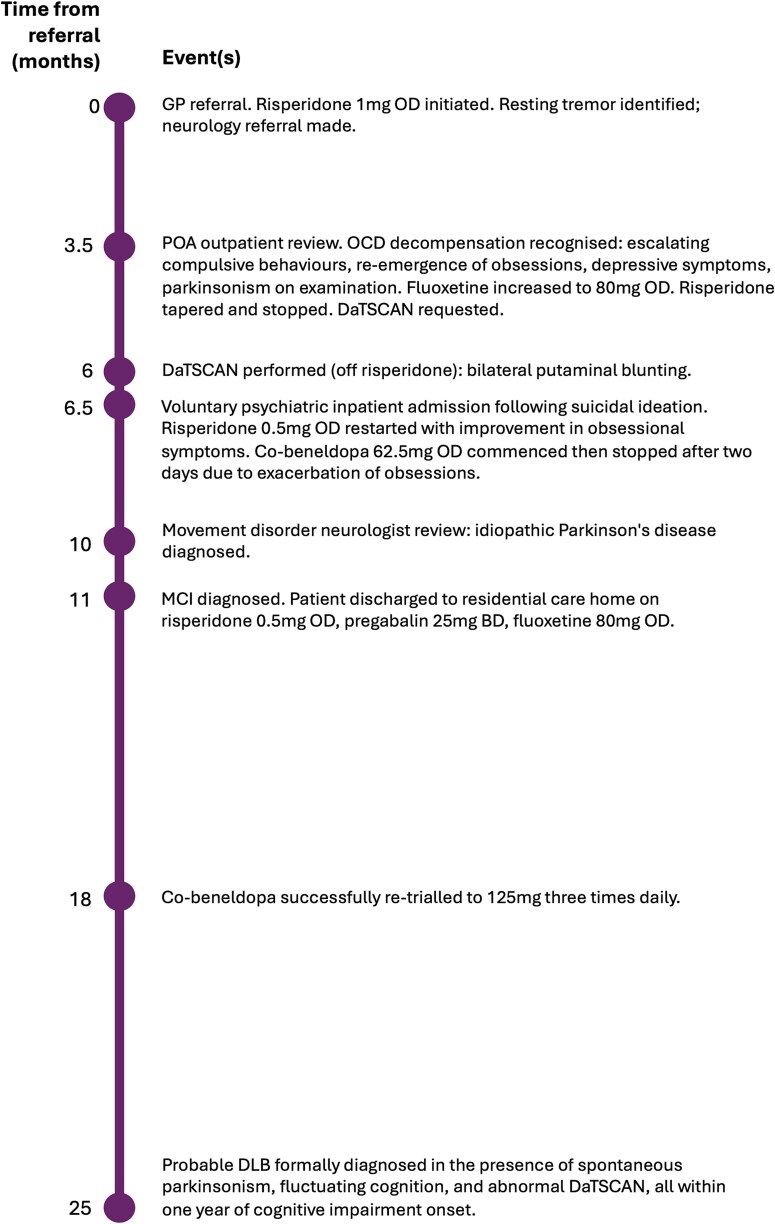
Timeline of significant events.

We describe a patient with chronic but stable OCD whose psychiatric, motor, cognitive and functional abilities deteriorated over one year (Fig. 1), culminating in a probable DLB diagnosis (Table 1). Although initially diagnosed with idiopathic Parkinson’s disease, the one-year rule (requiring dementia onset to precede or co-emerge with parkinsonism [1]) was met, and the temporal co-emergence of parkinsonism, cognitive fluctuations, and abnormal DaTSCAN supported reclassification to probable DLB [1].

**Table 1:** Alignment of clinical findings with the consensus criteria for the diagnosis of dementia with Lewy bodies [4].

Diagnostic category	Criterion	Present in this case
*Core clinical features*	Fluctuating cognition with pronounced variations in attention and alertness	Yes—fluctuating attentional deficits, MMSE 25–29
	Recurrent visual hallucinations	No
	REM sleep behavior disorder	No
	Spontaneous parkinsonism	Yes—rest tremor, bradykinesia, rigidity
*Supportive clinical features*	Severe sensitivity to antipsychotic agents	Yes—exacerbation of parkinsonism with risperidone
	Postural instability and repeated falls	Yes
	Syncope or transient episodes of unresponsiveness	No
	Severe autonomic dysfunction	No—no postural hypotension or symptoms reported
	Hypersomnia	No
	Hyposmia	No
	Hallucinations in other modalities	No
	Systematized delusions	No—prominent obsessions but no delusional ideation
	Apathy, anxiety, and depression	Yes—prominent anxiety and depressive symptoms
*Indicative biomarkers*	Reduced DAT uptake on SPECT or PET	Yes—significant bilateral putaminal blunting
	Abnormal MIBG myocardial scintigraphy	Not performed
	Polysomnographic confirmation of REM sleep without atonia	Not performed
*Supportive biomarkers*	Relative preservation of medial temporal structures on CT/MRI	Not performed
	Reduced occipital activity on SPECT/PET	Not performed
	Prominent posterior slow-wave activity on EEG	Not performed

Although an evidence base for poDLB has recently emerged, this has largely referred to *de novo* psychiatric presentations [4]. This case is the first to describe decompensation of a stable, longstanding severe mental illness as a psychiatric prodrome of DLB. Only two prior cases identify OCD as a DLB manifestation [5], both of late onset and with considerable diagnostic uncertainty—symptoms were at points characterised as delusions rather than obsessions. In contrast, this patient’s symptoms were unequivocally obsessional and recognised as her established relapse signature.

This patient was diagnosed within a year of presentation, having parkinsonism at referral, consistent with the pattern in which early core clinical features predict a shorter interval to DLB diagnosis [4]. Earlier behavioural changes, including hoarding and compulsive spending in her early 50s, may represent an even longer prodromal course, consistent with the extended disease trajectories seen in isolated RBD. Clinicopathological studies have reported psychiatric symptoms emerging in the 50s in individuals later confirmed to have Lewy body pathology [6].

This case highlights the complexity of managing poDLB in routine practice. Risperidone cessation was likely implicated in the inpatient admission, illustrating that although neuroleptic sensitivity in DLB is well documented [7], the severity of some psychiatric presentations makes their use unavoidable. Antipsychotic prescription can obfuscate cognitive, motor and neuroimaging findings [8], restricting poDLB detection; importantly, the DaTSCAN in this case was acquired off risperidone, mitigating this confound.

This case illustrates the therapeutic trade-offs patients and clinicians face in DLB management. This patient prioritised OCD treatment over motor symptoms, acknowledging short-term motor deterioration; individual risk thresholds influencing such trade-offs may be explored through stated preference surveys [9]. The successful re-introduction of co-beneldopa 18 months after initial cessation suggests that drug sensitivity may evolve with disease progression, and previous intolerance should not preclude cautious re-trial where motor symptoms significantly impact quality of life.

Strengths include multidisciplinary input, detailed longitudinal psychiatric history, and DaTSCAN imaging which mitigated diagnostic confounds. Limitations include the absence of formal psychometric testing and detailed biomarker characterisation, though neither would have increased diagnostic certainty within the prodromal DLB framework [3].

This case highlights the need for heightened vigilance for neurodegenerative disease in older patients with decompensating primary psychiatric illness. In the United Kingdom, where most dementia diagnoses are made within psychiatry-aligned services, functional mental illness settings represent an underutilised opportunity for earlier DLB detection that policymakers should consider; toolkits supporting DLB recognition may be adapted for use in these settings [10]. For researchers, prospective studies of poDLB should consider decompensation in pre-existing illness alongside *de novo* presentations.
